# Subnormal Cytokine Profile in the Tear Fluid of Keratoconus Patients

**DOI:** 10.1371/journal.pone.0016437

**Published:** 2011-01-27

**Authors:** Albert S. Jun, Leslie Cope, Caroline Speck, Xiaojun Feng, Seakwoo Lee, Huan Meng, Abdel Hamad, Shukti Chakravarti

**Affiliations:** 1 Department of Ophthalmology, Johns Hopkins School of Medicine, Baltimore, Maryland, United States of America; 2 Department of Oncology, Johns Hopkins School of Medicine, Baltimore, Maryland, United States of America; 3 Department of Psychological and Brain Sciences, Johns Hopkins School of Arts and Sciences, Baltimore, Maryland, United States of America; 4 Department of Medicine, Johns Hopkins School of Medicine, Baltimore, Maryland, United States of America; 5 Department of Pathology, Johns Hopkins School of Medicine, Baltimore, Maryland, United States of America; 6 Department of Cell Biology, Johns Hopkins School of Medicine, Baltimore, Maryland, United States of America; University of Reading, United Kingdom

## Abstract

Keratoconus, historically viewed as a non-inflammatory disease, is an ectatic corneal disorder associated with progressive thinning of the corneal stroma. Recently, a few inflammatory mediators have been reported to be elevated in the tear fluid of keratoconus patients. Consequently, we investigated a wide range of inflammation regulating cytokines in the tears and sera of keratoconus and control subjects. Interleukin (IL)-1β, IL-4, IL-6, IL-10, IL-12, IL-13, IL-17, interferon (IFN)-γ, chemokine C-C motif ligand 5 (CCL5) and tumor necrosis factor (TNF)-α were tested in tear samples and sera of keratoconus and control individuals by multiplex immuno-bead assays. Selected cytokines were further tested by standard ELISA on pooled tear samples. All cytokines in the sera were generally low, with no significant changes between keratoconus and control subjects. However, in tear fluids, clear differences were detected between the two groups. These differences include increased IL-6, and decreased IL-12, TNF-α, IFN-γ, IL-4, IL-13 and CCL5 in keratoconus compared to control tear fluids. The decreases in IL-12, TNF-α and CCL5 were statistically significant, while the IL-13 decrease was statistically significant in the severe keratoconus group only. IL-17 could not be detected by multiplex immuno-bead assay, but showed an increase in keratoconus by conventional ELISA on a limited number of pooled tear samples. Our findings confirm increased IL-6, but dispute earlier reports of increased TNF-α, and suggest a cytokine imbalance in keratoconus disrupting corneal homeostasis. Moreover, an increase in IL-17 suggests tissue degenerative processes at work, contributing to the thinning and weakening of the corneal connective tissue in keratoconus.

## Introduction

Keratoconus is an ectatic corneal disease associated with progressive thinning of the corneal stroma, and a protruding, cone-shaped cornea that produces astigmatism and myopia. Keratoconus is a leading cause of corneal transplantation, affecting 1 in 2000 individuals with a mean age of onset at 15.4 years [1], [2], [3], [4]. The disorder typically progresses until the third to fourth decade of life [3], and the factors that determine the progression or stabilization of the disease are not well characterized or understood.

Although the etiology of keratoconus is poorly understood, it is traditionally viewed as a non-inflammatory corneal thinning disease [1]. Accordingly, cellular infiltration and vascularization are not clinically apparent in keratoconus. However, keratoconus has been linked to atopy since the 1950s [5], with further support coming from later studies that reported elevated levels of immunoglobulin (Ig) E in the sera of keratoconus patients [6], [7]. Recent studies have suggested pro-inflammatory factors as key to keratoconus pathogenesis based on their findings of elevated interleukin (IL)-6, tumor necrosis factor (TNF)-α and matrix metalloproteinase (MMP)-9 in the tear fluid of keratoconus patients [8], [9]. Moreover, increased binding of IL-1 by keratoconus corneal fibroblasts has led another group to suggest a role for inflammation in the onset or progression of keratoconus as well [10].

Despite these initial findings of specific changes in inflammatory cytokines, there are no studies that have examined a range of cytokines to determine whether keratoconus is associated with an imbalance in the repertoire of cytokines that regulate inflammatory and immune responses driven by subsets of T-helper cells, T_H_1, T_H_2, and T_H_17 in the corneal environment. To begin addressing this question, we quantified T_H_1 cytokines (IL-12, IFN-γ and TNF-α), T_H_2 cytokines (IL-4, IL-10 and IL-13), the T_H_17 representative cytokine IL-17, and other inflammatory cytokines/chemokines (IL-1β, IL-6, and RANTES or CCL5) in tear fluids and serum samples of keratoconus patients and control subjects. Although this initial study is based on a relatively small sample size, decreases in specific cytokines suggest down regulations of both pro-inflammatory and immunoprotective responses to play a role in this disease.

## Results

### Demographics

A set of 18 keratoconus (KC) and 11 controls were used for the multiplex cytokine analyses on tear fluids and sera (Table 1). Two (11%) of the KC patients were graded as mild (steepest K<45D), 6 (33%) as moderate (45D ≤steepest K≤52D), and 10 (56%) as severe (steepest K>52D). Using the Mann-Whitney test, there was a statistically significant (p = 0.02), 10 year difference in the mean age between the controls and the keratoconus subjects, who were on average 33 and 43 years old, respectively. Nine of the KC and four of the control subjects wore contact lenses. Due to their disease status, keratoconus patients normally wear hard contact lenses, while unaffected subjects wear soft contact lenses. Although matching by contact lens use would not *per se* remove this difference, to determine if contact lens use had an effect, irrespective of disease status, we fit a multivariate linear model to determine the effects of contact lens wear on tear fluid cytokine levels after adjusting for the age of the subjects. Levels of all cytokines tested were higher in the 13 contact lens users when compared to non-contact lens users, and while the differences were moderate for some of the cytokines, they were not statistically significant except for IL-4 (Table S1). The average time since diagnosis of keratoconus was 16±14 (mean ± SD) years (Table 1). Three of the 18 keratoconus patients had a medical history including atopy. Tear fluid samples were collected between 9 am and 4 pm, with no statistically significant differences in collection times between control and keratoconus subjects. We also noted that the keratoconus group was ethnically more diverse than the control group. Our recent efforts are geared towards collecting tear samples from appropriately matched control subjects, and when applicable to collect tear fluids from the unaffected eye of keratoconus patients as well.

**Table 1:** Keratoconus and control samples for immuno-bead multiplex ELISA.

Sample			Age	Gender		Contact lens	Diagnosis
Number	Group	Severity	(years)	(M/F)	Race	use (years)	(years)
1	KC	Severe	42	M	Black	None	?
2	KC	Severe	44	F	Native American	30.0	32
3	KC	Mild	52	F	White	Intolerant	?
4	KC	Severe	34	F	White	None	9
5	KC	-	76	F	Black	42.0	42
6	KC	Mild	38	M	Asian	Yes	10
7	KC	-	45	M	White	None	7
8	KC	Severe	30	M	Black	Yes	?
9	KC	Severe	31	M	Native American	None	3
10	KC	Severe	27	M	Asian	None	6
11A	KC	Severe	27	M	Native American	None	10
12	KC	Severe	35	M	White	None	9
13	KC	Mild	32	F	Native American	5.0	8
14A	KC	Mild	61	F	White	20.0	25
15	KC	-	61	M	White	None	48
16A	KC	Mild	40	M	White	24.0	7
17	KC	Mild	56	F	Black	Yes	22
18	KC	Mild	53	M	White	27.0	8
Mean			44			24.7	16
SD			14			12.2	14
1	Control	N/A	22	F	White	10.0	N/A
2	Control	N/A	50	F	White	None	N/A
3	Control	N/A	51	M	White	None	N/A
4	Control	N/A	30	M	Native American	None	N/A
5	Control	N/A	40	M	Asian	None	N/A
6	Control	N/A	31	F	White	10.0	N/A
7	Control	N/A	24	M	White	9.0	N/A
8	Control	N/A	30	M	White	None	N/A
9	Control	N/A	31	F	White	None	N/A
10	Control	N/A	30	F	White	None	N/A
11	Control	N/A	24	F	White	10.0	N/A
Mean			33			9.8	
SD			10			0.5	

A; atopy, KC: keratoconus, M; male, F: female, SD: 1 standard deviation, N/A: not applicable,

- Unable to obtain a keratometry measurement for severity due to irregular curvature of patient's cornea.

A second set of samples comprising 29 KC and 38 control samples were collected for performing individual ELISA assays for validation purposes (Table S2). In this set, the control subjects were matched more closely to the KC group for age and ethnicity. The mean ± SD ages were 38±10 and 40±12 years, for KC and control subjects, respectively. Some of the KC subjects wore contact lenses, while the control subjects for this group were not contact lens users (Table S2). Of the keratoconus and control cases (Table S2), atopy was reported for six and four of the individuals, respectively.

### Multiplex cytokine analysis of keratoconus and control subjects

**Figure 1:**
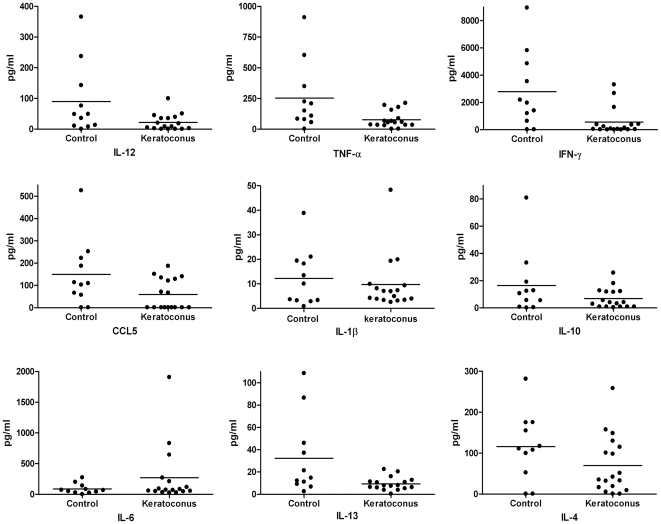
Cytokine measurements in tear fluids by a multiplex immuno-bead assay. Each cytokine was measured in individual subjects. The horizontal bar indicates the mean value for each group. After correcting for age and contact lens use, IL-12, TNF-α, and RANTES/CCL5 were significantly lower in KC than in controls. Statistical analyses of the data are shown in Tables 2 and 3.

To investigate the relationship between disease status and cytokine levels, we performed a linear multivariate correlation study of natural log transformed cytokine levels and disease, after correcting for age and contact lens use, as covariates. The results were reported as significant in Table 2, only if disease was a significant independent predictor of cytokine levels. All cytokines tested by multiplex immuno-bead assay, except IL-17, were detectable in tear samples. Table 2 shows median, 25^th^ and 75^th^ percentile values of tear fluid cytokines of all keratoconus and control subjects. Overall, we found IL-6 levels were 3 fold elevated (based on mean values) in keratoconus samples compared to control subjects (Table 2 and Figure 1), confirming a previous report of increased IL-6 in keratoconus [8]. The increase in IL-6 did not reach statistical significance due to the variation in levels between subjects. IL-1β was essentially unchanged, while the remaining cytokines showed decreased levels in our keratoconus samples. After subtracting the effects of age and contact lens use, the decreases in T_H_1 related cytokines IL-12 (p = 0.03), TNF-α (p = 0.04) and CCL5 (p = 0.05) were statistically significant. The decrease in TNF-α seen here, contradicts an earlier observation of increased TNF-α in keratoconus. Statistical differences in cytokine levels were also analyzed after stratifying the keratoconus group into mild/moderate (steepest K≤52D) and severe (steepest K>52 D) groups. No significant differences were evident when the mild to moderate disease was compared to the controls (analysis not shown). The comparison of the data from severe KC to that of controls is shown in Table 3. Despite the resulting decrease in sample size, IL-12, TNF-α and CCL5 showed even stronger effects in severe keratoconus. The IFN-γ decrease in keratoconus compared to controls was significant by Mann-Whitney testing (p = 0.02, analysis not shown), but not by multivariate analysis, where the large variance of the measurements appeared to diminish its significance (Table 3). Of the T_H_2-related cytokines, the decrease in IL-13 was statistically significant in severe keratoconus versus control subjects. The multiplex immuno-bead assay could not detect IL-17 in control or keratoconus tear samples. Atopy was not considered as a testable variable since it was detected in similar numbers in keratoconus and control individuals. We found no correlation (Methods) between the time of tear fluid collection and cytokine levels (not shown).

**Table 2:** Tear-fluid cytokine concentrations in keratoconus and control subjects.

	Control (C)		Keratoconus (KC)		Fold	
	Median, quartiles^a^	Mean ± SD	Median, quartiles^a^	Mean ± SD	change	*p* –
Cytokine	pg/ml	pg/ml	pg/ml	pg/ml	KC/C^b^	value^c^
IL-1β	10 [3.2, 19.4]	9.6±7.9	6.9 [3.6, 9.6]	8.3±6.7	0.88	0.87
IL-4	111.2 [52.6, 175.1]	116.1±81.9	38.7 [12.8, 122.7]	69.6±70.7	0.6	0.43
IL-6	66.8 [27.4, 140.4]	89.5±83.5	72.2 [48.9, 241]	282.7±467.7	3.2	0.17
IL-10	10.6 [0.7, 19.1]	16.5±23.5	4 [0.9, 12.1]	6.9±7.3	0.4	0.4
IL-12	48.6 [10.6, 142.8]	89.8±115.8	8.4 [1.6, 37.1]	21.8±26.6	0.24	0.03
IL-13	14.7 [9.1, 46]	32.3±33.5	7.8 [5.7, 12.1]	11.2±5.9	0.35	0.11
IFN-γ	1969 [643, 4859]	2783±2778.7	76 [19.3, 401.6]	557.1±1004.3	0.2	0.2
CCL5	109.4 [57, 222]	111.3±86.3	1.2 [1.2, 131.7]	59.4±68.9	0.53	0.05
TNF-α	150 [80.5, 349.5]	252.6±275.3	54.6 [34.4, 122.3]	77.1±669	0.3	0.04

a 25^th^ and 75^th^ percentile.

b fold change in mean values.

c *p* value based on a multivariate linear model after correcting for age and contact lens use covariates.

**Table 3:** Tear-fluid cytokine concentrations in severe (K>52D) keratoconus and control subjects.

	Control (C)	Keratoconus (KC)	Fold change	
Cytokine	Mean ± SD, pg/ml	Mean ± SD, pg/ml	KC/C	*p* a-value
IL-1β	12.19±11.56	7.46±5.58	0.61	0.83
IL-4	116±81.8	33.48±49.74	0.3	0.31
IL-6	89.64±83.36	254±312.41	2.83	0.27
IL-10	16.46±23.5	7.05±9	0.41	0.17
IL-12	90.1±115.57	12.57±15.25	0.14	0.007
IL-13	32.34±35.1	8.79±6	0.27	0.04
IFN-γ	2780.6±2781.3	543.83±946.28	0.2	0.133
CCL5	149±149.4	25.8±48.1	0.18	0.013
TNF-α	253±275	55.06±67.72	0.22	0.04

a *p* value based on a multivariate linear model after correcting for age and contact lens use covariates.

In the sera, there were no statistically significant differences between keratoconus patients and control subjects for the cytokines tested (Table 4). IL-10, IL-12 and IL-13 could not be detected in any of the sera tested, while IL-1β, IL-4, IL-6 and TNF-α were present at very low levels, and CCL5 was clearly detected. Thus, the cytokine changes detected in the tear film are likely a reflection of localized events in the eye and not systemic changes.

**Table 4:** Serum cytokine concentrations in keratoconus and control subjects.

	Control (C)	Keratoconus (KC)	Fold change	
Cytokine	Mean ± SD, pg/ml	Mean ± SD, pg/ml	KC/C^a^	*p* b-value
IL-1β	0.18±0.15	0.14±0.11	0.8	0.71
IL-4	0.09±0.06	0.11±0.08	1.2	0.61
IL-6	1.77±3.66	1.39±2.37	0.8	0.31
IL-10	ND	ND	N/A	-
IL-12	ND	ND	N/A	-
IL-13	ND	ND	N/A	-
IFN-γ	27.49±9.73	25.84±25.38	0.9	0.4
CCL5	100.70±55.61	93.19±58.0	0.9	0.73
TNF-α	1.46±1.71	1.57±1.42	1.1	1.0

a Fold change in mean cytokine level.

b *p* value based on Mann-Whitney test. ND: Not detectable. All readings were below detection levels.

N/A: not applicable.

### Measurements of selected cytokines in tear fluid by standard ELISA

**Figure 2:**
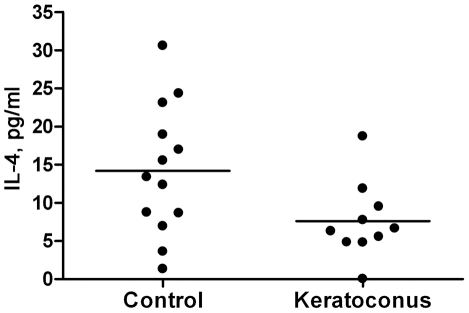
Decrease in IL-4 measured by standard ELISA. Samples shown in Table S2 were pooled into 13 control and 10 keratoconus samples (Methods). Each ELISA value is an average of two measurements. The decrease in IL-4 in the keratoconus pool (7.6±5 pg/ml), compared to controls (14.2±8.6 pg/ml) was statistically significant (p≤0.05, Mann-Whitney test). The horizontal bar indicates the mean value for each group.

**Figure 3:**
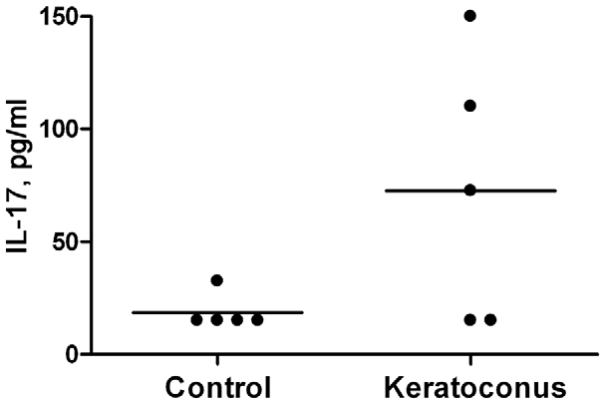
Increase in IL-17 measured by standard ELISA. Five pools of keratoconus and control subjects (Table S2) were used. Each ELISA value is an average of two measurements. The increase in IL-17 in keratoconus samples (72.5±59.2 pg/ml) compared to controls (18.5±7.8 pg/ml), was statistically significant (p≤0.05, Mann-Whitney test). The horizontal bar indicates the mean value for each group.

The multiplex data implicate reductions in T_H_1 (IL-12, TNF-α, IFN-γ), and possibly T_H_2 cytokines (IL-4 and IL-13) in keratoconus. To further explore these possibilities, we performed standard individual ELISAs for selected cytokines. In general, the conventional, standard ELISA (high sensitivity) is less sensitive than multiplex immuno-bead assays. Therefore, it was necessary to pool the tear samples from different KC and control subjects (Table S2) for these ELISA experiments. Based on the commercial availability of high sensitivity ELISA kits, we selected TNF-α and IFN-γ as T_H_1, IL-4 as T_H_2 and IL-17 as T_H_17 representative cytokines. We found the sensitivity of the TNF-α kit to be too low for TNF-α detection in the tear fluids by ELISA (data not shown). IFN-γ was detectable and showed a decrease in keratoconus compared to pooled control samples but without statistical significance (p = 0.5, data not shown). We detected 14.2±8.6 pg/ml (mean ± SD) of IL-4 in the control and 7.6±5 pg/ml in the KC pool (Figure 2); the IL-4 decrease in KC was statistically significant (p = 0.04), confirming the multiplex immuno-bead assay results (Figure 1, Tables 2 and 3). IL-17 was detectable in one control and 3 keratoconus pools in one experimental set. All negative values in that set, being below the level of detection, were set at the minimum detection level for this ELISA kit. Based on these limited data (Figure 3), there was a trend towards increased IL-17 (72.5±59.2 pg/ml) in keratoconus tear fluids compared to control samples (18.5±7.8 pg/ml).

## Discussion

In this study, we investigated cytokines in the sera and tear fluids of keratoconus and control individuals to determine if altered inflammatory response is a factor contributing to the etiology of keratoconus. Of the cytokines measured in the serum, most were at very low levels and none showed significant differences between patients and control individuals. This is consistent with the idea that keratoconus is not associated with major systemic inflammation. However, our results indicate perturbations in immune related homeostasis in the tear film and the corneal microenvironment of keratoconus patients.

The published data on actual baseline cytokine concentrations in tear fluids is limited, and quite variable. To compare our findings to those of others, we examined the literature and found only a few instances where tear fluids were assessed for multiple cytokines. Since only a limited amount of tear fluid is obtainable without stimulation, measuring multiple cytokines is challenging. Li and coworkers used a solid phase antibody protein array-based method to estimate the relative amounts of tear fluid cytokines in Sjögren's syndrome, without reporting actual levels of cytokines [11]. A recent study used an immuno-bead based multiplex system to obtain baseline levels of 30 different cytokines and chemokines [12]. Others have examined isolated cytokines by conventional sandwich ELISA and reported concentrations of IL-6, TNF-α and IL-10 in control and keratoconus subjects [8], [13]. Our immuno-bead based assay, on average showed high levels of IL-4, IL-6, IL-12, IFN-γ, TNF-α and CCL5 in the tear samples of control subjects. Our baseline findings for IL-6 and TNF-α by multiplex assays were 5-10 times higher than the reported single ELISA measurements. However, our assay yielded baseline ranges for IL-10, IL-13, CCL5 and IL-6 that were within the range reported by the recent immuno-bead based study [12]. On the other hand, our estimates of TNF-α and IFN-γ were higher, and that of IL-1β was lower than that reported in the earlier study [12]. Since our immuno-bead assay kit is different from that used in the other study, some of this difference may arise from different primary and secondary antibodies. In addition to the variation seen between different studies, the range for each cytokine within a given study, including ours, is very large. Some of this variability could be due to individual-to-individual variation in cytokine levels and the extent of tearing even though samples are collected without direct stimulation.

Comparing tear fluid cytokines in the control and keratoconus subjects, we noted an increase in IL-6 in keratoconus, in agreement with earlier reports [8], [13]. In addition, we identified significant decreases in IL-12 and TNF-α. The latter observation disagrees with an earlier report of a small increase in TNF-α of keratoconus tear fluids. The possible reasons for this difference are, 1) use of different antibody-based assays, 2) actual TNF-α levels detected in the other study is very close to the lower limit of TNF-α detection by the conventional ELISA kit used in that study, and 3) differences in patient population between the two studies. IL-12 promotes the differentiation of T_H_1 cells; its decrease in keratoconus is consistent with decreases in two signature T_H_1 cytokines, IFN-γ and TNF-α. In severe keratoconus, IL-12 and TNF-α decreases were more pronounced and this may play a role in increased IL-17 and associated tissue degenerative processes. Both IL-4 and IL-13 cytokines were also reduced in keratoconus, and the decrease in IL-4, as measured by conventional ELISA, was statistically significant. IL-4 is a key T_H_2 cell differentiation cytokine, and both IL-4 and IL-13 play crucial roles in amplification of the T_H_2 response through up regulation of STAT5, STAT6 and GATA3 transcription [14]. Thus, decreases in IL-4 and IL-13 suggest that T_H_2 responses may be dampened in keratoconus. IL-4 is associated with allergic response and promotes the synthesis of IgE [15]. Based on the reported link between atopy and keratoconus, it is conceivable that IL-4 levels would be higher in the keratoconus group. However, the number of patients with a history of atopy in our samples was extremely low. Thus, separate from atopy, the decrease in IL-4 we see may be pertinent to the pathophysiology of keratoconus itself. How do our tear fluid cytokine observations in keratoconus compare to other ocular surface diseases? A study reported increases in IL-1β, IL-2, IL-5, IL-6, IL-12, IL-13, in seasonal allergic conjunctivitis (SAC), vernal keratoconjunctivitis (VKC) and atopic keratoconjunctivitis (AKC), while IL-4, IFN-γ and IL-10 levels were increased in SAC and VKC compared to controls [16]. These observations are certainly different from the cytokine profile we see in keratoconus. Thus, the broad decrease in inflammatory cytokines reported in our study may indeed be keratoconus disease-specific.

Decreased IL-4 in keratoconus may have broad immune and non-immune consequences [17] that should be considered in the context of corneal thinning and abnormal stromal extracellular matrix (ECM). IL-4 and IL-4 receptor engagement through the JAK-STAT and the IRS1/2 pathways regulate cell proliferation and tissue homeostasis [17]. Dermal fibroblasts stimulated with IL-4 and IL-13 up regulate production of collagens type I and III [18]. IL-4 mediated induction of STAT-6 pathway in intrahepatic cells has been shown to induce collagen synthesis [19]. While its implication in hepatic disease is over-induction of STAT-6, increased collagen, and fibrosis, reduction of normal levels of IL-4 in the corneal environment may tip the balance toward poor stromal keratocyte cell survival, oxidative stress, altered collagen and ECM stability associated with keratoconus [20], [21], [22], [23].

*In vitr*o and *in vivo* studies indicate reciprocal interactions between IFN-γ and IL-17, and between IL-4 and IL-17, and that these cytokines restrict undue amplification of the T_H_17 response [24]. It is possible that in the pathogenesis of keratoconus, decrease and dysregulation of several cytokines and growth factors encourage aggravated T_H_17 response, production of metalloproteinases and tissue damage. Furthermore, TGF-β and IL-6 levels regulate differentiation of Treg and T_H_17 subsets of T cell [25]. TGF-β members are present in all ocular tissues [26], [27], [28], and, by immunohistochemistry we detected stronger immunostaining for total TGF-β in the epithelial layer (our unpublished observations) of keratoconus corneas. Moreover, IL-6 levels were increased in the tear fluid of keratoconus patients as indicated in this study and reported earlier [8]. Indeed, whether relative TGF-β and IL-6 levels are altered sufficiently in keratoconus ocular tissues are not known at this time. On testing IL-17 by the multiplex immuno-bead assay, we could not detect it in the tear fluid. However, using an IL-17 ELISA kit, we detected higher levels of IL-17 in 3 of the five patient pools in one set of samples. An increasing number of studies link IL-17 to autoimmune and inflammatory diseases such as rheumatoid arthritis, lupus, asthma and psoriasis [29]. IL-17 is produced primarily by T_H_17 subset T lymphocytes, while IL-17 receptors are found in a broad array of cell types including fibroblasts and myofibroblasts [30], [31], [32]. Multiple studies are beginning to link IL-17 mediated induction of fibroblasts and myofibroblasts and the production of tissue degrading proteases and cytokines [31], [33], [34], [35]. Recent studies have also linked IL-17A to ocular pathogenesis of Sjögren's Syndrome [36]. Desiccating stress in dry eye disease appears to also involve increased MMP-9 and T_H_17 inflammation [37], [38], [39]. In dry eye, IL-17 promoting cytokines have been shown to be produced by isolated T cells in the conjunctiva and the cornea [37]. In our hands quantitative reverse transcriptase polymerase chain reaction on total RNA from scraped corneal epithelium failed to detect IL-17A. Additional immunohistology of conjunctiva impressions will be needed to further address the source of IL-17 producing cells and the validity of an IL-17 mediated inflammation and tissue damage in keratoconus. Two recent studies performed proteomic analyses on the tear fluids of unaffected individuals and keratoconus patients. In one the authors reported down regulations in lactoferrin and immunoglobulin kappa chains [40]. Using a cytokine antibody array, the second study [41] found a decrease in TNF-α in agreement with our findings here. This study reported a slight increase in TGF-β2 and β3, which would conform to our speculation that conditions in keratoconus may favor aggravated Th17 response.

In summary, keratoconus is a complex heterogeneous disease in which its altered corneal structure and functions may be related to multiple factors. Our data suggest that instead of a simple increase in pro-inflammatory cytokines, there may be a complex imbalance between pro-inflammatory and anti-inflammatory cytokines, and possibly aggravated T_H_17 response leading to altered epithelial and stromal functions. Clearly, it is important to confirm the observed cytokine changes in a larger patient population. To gain insights into the immunological processes that may contribute to these cytokine changes it is important to track down the cellular sources of these changes. While historically many studies have focused on the cornea itself, the tear fluid changes implicate the conjunctiva and the lacrimal gland as having some paracrine effects in this disease.

## Materials and Methods

### Samples

All patients and normal subjects provided written informed consent according to a protocol used in the current study that was approved by the Johns Hopkins Institutional Review Board. Inclusion criterion for participation in the study was presence of keratoconus as determined by clinical examination and corneal modeling (Pentacam, Oculus, Lynnwood, WA). Participants completed a comprehensive questionnaire designed to collect information on quality of life, contact lens use, past medical history, and family history. For the multiplex cytokine analysis (Table 1) we used 18 patients (61% males, mean age ± standard deviation (SD) = 43±13 years) and 11 control (45% males, mean age ± SD = 33±10 years) tear samples. Whole blood was collected for serum extraction.

Conventional ELISA (Table S2) was performed for selected cytokines on pooled samples from an additional 29 keratoconus patients (76% male, mean age = 38±10) and 38 control individuals (52% male, mean age = 40±12). These samples were pooled to generate 13 control and 10 keratoconus pools. The keratoconus samples were pooled based on disease status (mild, moderate and severe).

Severity of keratoconus was graded by the steepest keratometry (K) measurement with <45 diopters (D) being mild, 45≤K≤52D being moderate, and severe >52D or not measurable. Ophthalmic exams consisted of best corrected visual acuity measurements, slit lamp biomicroscopy, and corneal topography using the Pentacam.

### Tear Analysis

An aliquot of 5 to 8 µl of tear fluid was collected from each eye of participants, provided that the eye had not undergone previous surgery. Tear samples were obtained by capillary flow using micropipettes with care taken to minimize reactive tear production. Samples were frozen to −80°C within one hour of collection. Tear samples were diluted 1∶25 in phosphate buffered saline (Quality Biological, Gaithersburg, MD) containing 1 mg/ml BSA to a final volume of 100 µl according to protocols supplied by the multiplex immuno-bead assay kit (Human Bio-Plex suspension array system, Biorad). The concentrations of cytokines (interleukin (IL)-1β, IL-4, IL-6, IL-10, IL-12, IL-13, interferon (IFN)-γ, tumor necrosis factor (TNF)-α, and IL-17) and the chemokine (CC-motif) ligand 5 (CCL5) were measured using a Bio-Plex Luminex 100 (Biorad).

Conventional enzyme-linked immunosorbent assay (ELISA) was used to detect levels of selected cytokines in pooled tear fluid samples diluted 1∶2. The following ELISA kits were used for IFN-γ (Cat# DIF50, Human IFN-gamma Quantikine ELISA Kit, R&D Systems), IL-4 (Cat# HS400, Human IL-4 Quantikine HS ELISA Kit, R&D Systems) and IL-17A (Cat# D1700, human IL-17 Quantikine ELISA Kit, R&D Systems). Based on the standard curves the detection sensitivity for IFN-γ, IL-4 and IL-17 were 8 pg/ml, 0.11 and 15 pg/ml, respectively.

### Serum Analysis

An aliquot of 10 ml blood was collected in a clot activating tube (Becton Dickinson, Franklin Lakes, NJ) and allowed to stand at room temperature one to two hours before spinning 15 minutes at 4°C. Serum aliquots were immediately transferred to −80°C for storage. Undiluted serum samples were assayed using the Bio-Plex Human Cytokine Assay kit, (Biorad, Hercules, CA) for IL-1β, IL-4, IL-6, IL-10, IL-12, IL-13, IL-17, IFN-γ, CCL5, TNF-α as described for tear fluid samples.

### Statistical Analysis

Chi-square tests were used to determine statistical significance for categorical variables between groups. Cytokine levels in keratoconus and control subjects were first compared using a 2-tailed, Mann-Whitney nonparametric test (Prism; GraphPad Software, San Diego, CA.), with a p-value≤0.05 considered to be statistically significant. To determine significant (p≤0.05) associations between disease status and cytokine changes, we fit a multivariate linear model using natural log transformed cytokine levels, with age in years, and contact lens use as covariates. These multivariate analyses were performed using the R statistical software (http://www.R-project.org). We found no correlation between cytokine level and time of tear fluid collection (Spearmen correlation, Prism; GraphPad) and did not include collection time as a variable in the multivariate analysis.

## Supporting Information

Table S1 **Cytokine concentrations in contact lens users and non-users**
(DOC)

Table S2 **Keratoconus and control samples for ELISA measurements of selected cytokines**
(DOC)

## References

[1] Krachmer JH, Feder RS, Belin MW (1984). Keratoconus and related noninflammatory corneal thinning disorders.. Surv Ophthalmol.

[2] Yue BY, Sugar J, Benveniste K (1984). Heterogeneity in keratoconus: possible biochemical basis.. Proc Soc Exp Biol Med.

[3] Rabinowitz YS (1998). Keratoconus.. Surv Ophthalmol.

[4] Olivares Jimenez JL, Guerrero Jurado JC, Bermudez Rodriguez FJ, Serrano Laborda D (1997). Keratoconus: age of onset and natural history.. Optom Vis Sci.

[5] Galin MA, Berger R (1958). Atopy and keratoconus.. Am J Ophthalmol.

[6] Kemp EG, Lewis CJ (1982). Immunoglobulin patterns in keratoconus with particular reference to total and specific IgE levels.. Br J Ophthalmol.

[7] Rahi A, Davies P, Ruben M, Lobascher D, Menon J (1977). Keratoconus and coexisting atopic disease.. Br J Ophthalmol.

[8] Lema I, Duran JA (2005). Inflammatory molecules in the tears of patients with keratoconus.. Ophthalmology.

[9] Lema I, Sobrino T, Duran JA, Brea D, Diez-Feijoo E (2009). Subclinical keratoconus and inflammatory molecules from tears.. Br J Ophthalmol.

[10] Fabre EJ, Bureau J, Pouliquen Y, Lorans G (1991). Binding sites for human interleukin 1 alpha, gamma interferon and tumor necrosis factor on cultured fibroblasts of normal cornea and keratoconus.. Curr Eye Res.

[11] Li S, Sack R, Vijmasi T, Sathe S, Beaton A (2008). Antibody protein array analysis of the tear film cytokines.. Optom Vis Sci.

[12] Carreno E, Enriquez-de-Salamanca A, Teson M, Garcia-Vazquez C, Stern ME (2010). Cytokine and chemokine levels in tears from healthy subjects..

[13] Lema I, Duran JA, Ruiz C, Diez-Feijoo E, Acera A (2008). Inflammatory response to contact lenses in patients with keratoconus compared with myopic subjects.. Cornea.

[14] Paul WE, Zhu J (2010). How are T(H)2-type immune responses initiated and amplified?. Nat Rev Immunol.

[15] Wills-Karp M, Finkelman FD (2008). Untangling the complex web of IL-4- and IL-13-mediated signaling pathways.. Sci Signal.

[16] Leonardi A, Curnow SJ, Zhan H, Calder VL (2006). Multiple cytokines in human tear specimens in seasonal and chronic allergic eye disease and in conjunctival fibroblast cultures.. Clin Exp Allergy.

[17] Nelms K, Keegan AD, Zamorano J, Ryan JJ, Paul WE (1999). The IL-4 receptor: signaling mechanisms and biologic functions.. Annu Rev Immunol.

[18] Bhogal RK, Bona CA (2008). Regulatory effect of extracellular signal-regulated kinases (ERK) on type I collagen synthesis in human dermal fibroblasts stimulated by IL-4 and IL-13.. Int Rev Immunol.

[19] Aoudjehane L, Pissaia A, Scatton O, Podevin P, Massault PP (2008). Interleukin-4 induces the activation and collagen production of cultured human intrahepatic fibroblasts via the STAT-6 pathway.. Lab Invest.

[20] Chwa M, Atilano SR, Reddy V, Jordan N, Kim DW (2006). Increased stress-induced generation of reactive oxygen species and apoptosis in human keratoconus fibroblasts.. Invest Ophthalmol Vis Sci.

[21] Kenney MC, Brown DJ (2003). The cascade hypothesis of keratoconus.. Cont Lens Anterior Eye.

[22] Fini ME, Yue BY, Sugar J (1992). Collagenolytic/gelatinolytic metalloproteinases in normal and keratoconus corneas.. Curr Eye Res.

[23] Fukuchi T, Yue BY, Sugar J, Lam S (1994). Lysosomal enzyme activities in conjunctival tissues of patients with keratoconus.. Arch Ophthalmol.

[24] Steinman L (2007). A brief history of T(H)17, the first major revision in the T(H)1/T(H)2 hypothesis of T cell-mediated tissue damage.. Nat Med.

[25] Awasthi A, Murugaiyan G, Kuchroo VK (2008). Interplay between effector Th17 and regulatory T cells.. J Clin Immunol.

[26] Imanishi J, Kamiyama K, Iguchi I, Kita M, Sotozono C (2000). Growth factors: importance in wound healing and maintenance of transparency of the cornea.. Prog Retin Eye Res.

[27] Long Q, Chu R, Zhou X, Dai J, Chen C (2006). Correlation between TGF-beta1 in tears and corneal haze following LASEK and epi-LASIK.. J Refract Surg.

[28] Saika S (2006). TGFbeta pathobiology in the eye.. Lab Invest.

[29] Wynn TA (2005). T(H)-17: a giant step from T(H)1 and T(H)2.. Nat Immunol.

[30] Yagi Y, Andoh A, Inatomi O, Tsujikawa T, Fujiyama Y (2007). Inflammatory responses induced by interleukin-17 family members in human colonic subepithelial myofibroblasts.. J Gastroenterol.

[31] Cortez DM, Feldman MD, Mummidi S, Valente AJ, Steffensen B (2007). IL-17 stimulates MMP-1 expression in primary human cardiac fibroblasts via p38 MAPK- and ERK1/2-dependent C/EBP-beta, NF-kappaB, and AP-1 activation.. Am J Physiol Heart Circ Physiol.

[32] Sylvester J, Liacini A, Li WQ, Zafarullah M (2004). Interleukin-17 signal transduction pathways implicated in inducing matrix metalloproteinase-3, -13 and aggrecanase-1 genes in articular chondrocytes.. Cell Signal.

[33] Bamba S, Andoh A, Yasui H, Araki Y, Bamba T (2003). Matrix metalloproteinase-3 secretion from human colonic subepithelial myofibroblasts: role of interleukin-17.. J Gastroenterol.

[34] Rifas L, Arackal S (2003). T cells regulate the expression of matrix metalloproteinase in human osteoblasts via a dual mitogen-activated protein kinase mechanism.. Arthritis Rheum.

[35] Qiu Z, Dillen C, Hu J, Verbeke H, Struyf S (2009). Interleukin-17 regulates chemokine and gelatinase B expression in fibroblasts to recruit both neutrophils and monocytes.. Immunobiology.

[36] Katsifis GE, Moutsopoulos NM, Wahl SM (2007). T lymphocytes in Sjogren's syndrome: contributors to and regulators of pathophysiology.. Clin Rev Allergy Immunol.

[37] De Paiva CS, Chotikavanich S, Pangelinan SB, Pitcher JD, Fang B (2009). IL-17 disrupts corneal barrier following desiccating stress.. Mucosal Immunol.

[38] Chauhan SK, El Annan J, Ecoiffier T, Goyal S, Zhang Q (2009). Autoimmunity in dry eye is due to resistance of Th17 to Treg suppression.. J Immunol.

[39] Zheng X, de Paiva CS, Li DQ, Farley WJ, Pflugfelder SC (2010). Desiccating Stress Promotes Th17 Differentiation by Ocular Surface Tissues through a Dendritic Cell-Mediated Pathway.. Invest Ophthalmol Vis Sci.

[40] Lema I, Brea D, Rodriguez-Gonzalez R, Diez-Feijoo E, Sobrino T (2010). Proteomic analysis of the tear film in patients with keratoconus.. Mol Vis.

[41] Pannebaker C, Chandler HL, Nichols JJ (2010). Tear proteomics in keratoconus.. Mol Vis.

