# Supplementary material for: Subnormal Cytokine Profile in the Tear Fluid of Keratoconus Patients
Source: PLoS One. 2011 Jan 27;6(1):e16437. doi: 10.1371/journal.pone.0016437 (PMC3029330; doi:10.1371/journal.pone.0016437)
Supplement: Table S1 — Cytokine concentrations in contact lens users and non-users (DOC) [file pone.0016437.s001.doc]

**Table S1. Cytokine concentrations in contact lens users and non-users**

|  | Contact lens users | Non-users |  |
| --- | --- | --- | --- |
| Cytokine | Mean ± SD | Mean ± SD | *p*-value |
|  | pg/ml | pg/ml |  |
| IL-1b | 12.3 ± 13 | 10.6 ±10.3 | 0.75 |
| IL-4 | 119.5 ± 85.3 | 61.7 ±.03 | 0.026 |
| IL-6 | 287.7 ± 503.9 | 152.2 ± 245.1 | 0.42 |
| IL-10 | 26.6 ± 50.6 | 7.6± 9.3 | 0.13 |
| IL-12 | 117.7 ± 212.3 | 35 ± 60.4 | 0.12 |
| IL-13 | 38 ± 64.4 | 16 ± 21.4 | 0.17 |
| IFN-g | 2908.8 ±3265.6 | 1224.5 ± 1670 | 0.07 |
| CCL5 | 176.2 ± 212.2 | 62.1 ± 81.9 | 0.05 |
| TNF-a | 308.5 ± 457.1 | 102.3 ± 151 | 0.08 |
