# Supplementary material for: Subnormal Cytokine Profile in the Tear Fluid of Keratoconus Patients
Source: PLoS One. 2011 Jan 27;6(1):e16437. doi: 10.1371/journal.pone.0016437 (PMC3029330; doi:10.1371/journal.pone.0016437)
Supplement: Table S2 — Keratoconus and control samples for ELISA measurements of selected cytokines (DOC) [file pone.0016437.s002.doc]

**Table S2. Keratoconus and control samples for ELISA measurements of selected c**ytokines

| Sample  Number | Group | Severity | Age (years) | Gender (M/F) | Race | Contact lens | Diagnosis  (years) |
| --- | --- | --- | --- | --- | --- | --- | --- |
| 1 | KC | Severe | 32 | M | White | Yes | 17 |
| 2 | KC | Moderate | 59 | F | White | No | 5 |
| 3 | KC | Moderate | 27 | M | White | No | 1 |
| 4 | KC | Severe | 33 | M | White | No | 15 |
| 5 | KC | Moderate | 21 | M | White | No | 2 |
| 6 | KC | Mild | 31 | M | White | No | 1 |
| 7 | KC | Mild | 25 | M | Other | Yes | 1 |
| 8 | KC | Severe | 36 | F | White | No | 1 |
| 9 | KC | Severe | 49 | M | White | Yes | 28 |
| 10 | KC | Severe | 39 | M | White | No | 5 |
| 11 | KC | Severe | 37 | M | Black | Yes | 23 |
| 12 | KC | Severe | 34 | F | White | No | 8 |
| 13A | KC | Severe | 55 | F | White | Yes | 25 |
| 14 | KC | Severe | 34 | M | White | Yes | 13 |
| 15A | KC | Severe | 42 | M | Black | Yes | 4 |
| 16 | KC | Severe | 25 | M | Native American | Yes | 7 |
| 17A | KC | Severe | 45 | F | White | No | 10 |
| 18 | KC | Severe | 48 | M | White | Yes | 0.17 |
| 19 | KC | Severe | 49 | M | White | Yes | 12 |
| 20 | KC | Severe | 21 | M | Black | Yes | 5 |
| 21A | KC | Moderate | 54 | F | White | No | 3 |
| 22 | KC | Moderate | 48 | M | White | Yes | 0.17 |
| 23A | KC | Moderate | 29 | M | White | Yes | 7 |
| 24 | KC | Moderate | 34 | F | White | Yes | 0.04 |
| 25 | KC | Moderate | 46 | M | White | Yes | 12 |
| 26A | KC | Moderate | 45 | F | White | No | 10 |
| 27 | KC | Moderate | 31 | M | Asian | No | 1 |
| 28 | KC | Moderate | 34 | M | White | No | 13 |
| 29 | KC | Moderate | 49 | M | White | Yes | 12 |
| Mean |  |  | 38 |  |  |  | 8.32 |
| SD |  |  | 10 |  |  |  | 7.76 |
| 1 | Control | N/A | 29 | F | Native American | No | N/A |
| 2 | Control | N/A | 64 | M | White | No | N/A |
| 3 | Control | N/A | 31 | F | White | No | N/A |
| 4A | Control | N/A | 49 | F | Black | No | N/A |
| 5 | Control | N/A | 47 | F | White | No | N/A |
| 6 | Control | N/A | 42 | M | Asian | No | N/A |
| 7 | Control | N/A | 48 | F | White | No | N/A |
| 8 | Control | N/A | 38 | M | White | No | N/A |
| 9 | Control | N/A | 42 | M | Asian | No | N/A |
| 10 | Control | N/A | 51 | M | Asian | No | N/A |
| 11 | Control | N/A | 57 | F | Asian | No | N/A |
| 12 | Control | N/A | 65 | M | White | No | N/A |
| 13 | Control | N/A | 66 | F | White |  | N/A |
| 14A | Control | N/A | 26 | F | Native American | No | N/A |
| 15 | Control | N/A | 23 | M | White | No | N/A |
| 16 | Control | N/A | 23 | M | White | No | N/A |
| 17 | Control | N/A | 51 | F | White | No | N/A |
| 18 | Control | N/A | 28 | M | White | No | N/A |
| 19 | Control | N/A | 34 | M | White | No | N/A |
| 20A | Control | N/A | 34 | F | White | No | N/A |
| 21 | Control | N/A | 51 | F | White | No | N/A |
| 22A | Control | N/A | 42 | M | White | No | N/A |
| 23 | Control | N/A | 23 | M | White | No | N/A |
| 24 | Control | N/A | 47 | F | White | No | N/A |
| 25 | Control | N/A | 23 | M | White | No | N/A |
| 26 | Control | N/A | 35 | M | White | No | N/A |
| 27 | Control | N/A | 41 | F | Black | No | N/A |
| 28 | Control | N/A | 27 | F | White | No | N/A |
| 29 | Control | N/A | 40 | M | Asian | No | N/A |
| 30 | Control | N/A | 38 | M | Asian | No | N/A |
| 31 | Control | N/A | 35 | F | Asian | No | N/A |
| 32 | Control | N/A | 41 | F | White | No | N/A |
| 33 | Control | N/A | 53 | M | Black | No | N/A |
| 34 | Control | N/A | 27 | M | Native American | No | N/A |
| 35 | Control | N/A | 28 | F | White | No | N/A |
| 36 | Control | N/A | 51 | M | White | No | N/A |
| 37 | Control | N/A | 33 | F | White | No | N/A |
| 38 | Control | N/A | 43 | M | Black | No | N/A |
| Mean |  |  | 40 |  |  |  |  |
| SD |  |  | 12 |  |  |  |  |

A: Atopy, KC: keratoconus, M; male, F: female, SD: 1 standard deviation, N/A: not applicable
